# Critical care services in Bagmati province of Nepal: A cross sectional survey

**DOI:** 10.12688/wellcomeopenres.19932.2

**Published:** 2024-06-04

**Authors:** Diptesh Aryal, Subekshya Luitel, Sushila Paudel, Roshni Shakya, Janaki Pandey, Isha Amatya, Prashant Acharya, Suman Pant, Hem Raj Paneru, Abi Beane, Rashan Haniffa, Pradip Gyanwali

**Affiliations:** 1 Nepal Intensive Care Research Foundation, Kathmandu, Bagmati Province, Nepal; 2 IDOR - D'Or Institute for Research and Education, Rio de Janerio, Brazil; 3 Mahidol Oxford Tropical Medicine Research Unit, Faculty of Tropical Medicine, Mahidol University, Bangkok, Thailand; 4 Nepal Health Research Council, Kathmandu, Bagmati Province, Nepal; 5 Tribhuvan University Teaching Hospital, Kathmandu, Nepal; 6 National Intensive Care Surveillance - MORU, Colombo, Sri Lanka; 7 Centre for Inflammation Research, The University of Edinburgh, Edinburgh, Scotland, UK

**Keywords:** intensive care unit (ICU), high dependency unit, critical care services, critical care resources, critical care structure, critical care staffing, lower middle-income country, Nepal

## Abstract

**Background:**

This study aimed to assess the current status of critical care services in 13 districts of Bagmati Province in Nepal, with a focus on access, infrastructure, human resources, and intensive care unit (ICU) services.

**Methods:**

A cross-sectional survey was conducted among healthcare workers employed in 87 hospitals having medical/surgical ICUs across Bagmati Province. Data were collected through structured questionnaires administered via face-to-face and telephone interviews. Descriptive analysis was used for data analysis, involving frequencies and percentages.

**Results:**

From 87 hospitals, a total of 123 ICUs were identified in the province, providing 1167 beds and 615 functioning ventilators. The average ICU bed availability per 100,000 population was 19, ranging from 3.6 in Makwanpur to 33.9 in Kathmandu. Out of 13 districts, 95% of beds were concentrated in just four districts, while six had no ICU facilities. Of the available facilities, 69.9% were owned by private entities. One-to-one nurse-to-ventilated bed ratio was maintained by 63.4% of ICUs during daytime, and 62.6% at nighttime. Furthermore, 74.8% of ICUs had consultants trained in critical care medicine. While essential equipment availability was higher in Bagmati province, gaps existed in the availability of oxygen plants and isolation rooms. Similarly, many ICUs offered continuous medical education and cardiopulmonary resuscitation (CPR) training, but improvements were necessary in clinical audits, antibiotic stewardship programs, and research engagement.

**Conclusions:**

Disparities in critical care resources were evident across districts in Bagmati Province, highlighting the need for a balanced and decentralized approach to ensure equitable access to care. Although there were disparities, numerous ICUs were effectively carrying out multiple critical care procedures. This study suggests conducting a nationwide mapping of ICU resources, prioritizing infrastructure development, optimizing resource allocation, and establishing national protocols.

## Introduction

Critical care services are essential for the management of critically ill patients in low- and middle-income countries (LMICs). However, the capacity of these services in LMICs is often limited by inadequate infrastructure, shortage of human resources, and limited availability of care facilities ^1, 2^.

Reporting on the capacity of critical care services in LMICs is important for several reasons. Firstly, the burden of disease in LMICs is often higher due to a higher incidence of infectious and non-communicable diseases ^3^. Secondly, LMICs often have limited resources and are unable to provide the same level of care as high-income countries. Besides, in low- and middle-income countries, managing an anticipated surge of critically ill patients particularly during pandemics is a major concern. The estimates suggest that there is an availability of 0.1 to 2.5 intensive care unit (ICU) beds per 100,000 population in these countries ^4^. Thirdly, understanding the capacity of critical care services in LMICs is essential for the development of effective and sustainable interventions to improve the quality of care provided to critically ill patients. The capacity of critical care services in LMICs is often different from that in high-income countries ^4, 5^. In LMICs, there are often fewer critical care beds and a shortage of trained personnel. Additionally, the availability of advanced technology and specialized equipment is often limited. These limitations can have a significant impact on patient outcomes and the quality of care provided ^6^.

Studies documenting critical resources are scarce in LMICs. It is essential to understand the capacity of critical care services in LMICs in order to develop effective strategies to improve the quality of care provided to critically ill patients. Furthermore, mapping critical care services is a fundamental step to evaluating the quality of existing service provision and identifying priorities for research and quality improvement ^1^. The South Asian region comprises 26% of the world population, but only one (Sri Lanka) out of the eight countries in the South Asian Association for Regional Cooperation (SAARC) region has so far mapped ICU resources nationwide ^7^. In the context of Nepal, only one study has so far examined the availability of personnel, equipment, and technology in adult intensive care units within the Kathmandu Valley ^8^, but its findings may not be indicative of the overall situation at a provincial or national level.

Nepal, a low and middle-income country in South Asia, has a population of approximately 30 million, with a majority residing in rural areas ^9^. The healthcare system in Nepal operates on a federal model, consisting of three levels: federal, provincial, and local. At each level, the Ministry of Health and Population (MoHP) is responsible for the overall health policy formulation, planning, organization, and coordination. The Ministry of Health and Population (MoHP)’s 2017 guidelines classify hospitals based on the services they provide rather than bed count, categorizing them into community level (Health Posts or Community Health Units), Primary Hospitals, Secondary Hospitals, Tertiary Hospitals, and Academic or Super-specialty hospitals ^10^. According to these guidelines, primary hospitals at the local level offer basic health services, while secondary and tertiary hospitals at the provincial and federal levels provide specialty services, including intensive care. However, the MoHP’s Department of Health Services (DOHS) annual report for 2021/2022 did not follow this new classification. Instead, the report indicates that Nepal had 192 public hospitals, 188 primary healthcare centers (PHCCs), 3,755 health posts (HPs), and 2,155 non-public (private) facilities ^11^. Despite the presence of these facilities, geographical access remains uneven, with poor accessibility to primary facilities in the northern mountain region, decreased accessibility of secondary facilities with greater distance from district centers, and limited accessibility of tertiary facilities in most areas except developed zones like zonal centers ^12^. This uneven distribution of healthcare facilities, particularly those offering specialized services like intensive care, poses challenges in ensuring equitable access to healthcare for the Nepali population.

The MoHP's 2014 guidelines mandate that at least 5 percent of total hospital beds should be ICU beds, with one ventilator for every two ICU beds, and a nurse-to-patient ratio of 1:1 ^13^. However, most hospitals do not meet these ICU criteria ^14^. While there have been advancements in hospitals and ICUs over the past decade, there is a lack of comprehensive data or publications on critical care services ^15^. To our knowledge, since 2011, no surveys have assessed critical care services in Nepal. During the COVID-19 pandemic in 2020, MoHP data indicated that Nepal had 194 hospitals with ICU facilities, comprising 1,595 ICU beds and 840 ventilators ^16^. The pandemic put immense pressure on hospital capacities, necessitating changes in capacities and resources, yet detailed data on these changes is lacking. Therefore, this paper aims to address these gaps by examining the current state of critical care services in 13 districts of Bagmati Province.

Bagmati Province is one of the seven provinces in Nepal, which was formed following the implementation of a new constitution in 2015 ^17^. This province has a total population of 6,116,866, encompassing 13 districts, including the capital city Kathmandu ^9^. Remarkably, the province also has the maximum number of ICUs in comparison to other provinces which can help us generalize the basic scenario of the intensive care facilities in Nepal. Despite having a maximum number of ICUs, access to critical care services is uneven, as most healthcare facilities are concentrated in major cities rather than rural regions. Many health facilities still likely have inadequate infrastructure, often lacking essential equipment and supplies. Furthermore, there is a shortage of trained healthcare professionals capable of providing critical care. While some hospitals may offer a wide array of specialized services, many health centers struggle to provide even basic critical care, leading to delays in treatment and poor patient outcomes. The study will delve into critical areas such as access to services, infrastructure availability, the adequacy of human resources, and the range of ICU services offered, providing valuable insights into the state of critical care in Nepal. This study will help understand the current capacity, which can aid in planning guidelines and preparing for emergencies in the future.

## Methods

A cross-sectional survey of ICUs was conducted across Bagmati Province, Nepal. Ethical clearance for the study was obtained from the Ethics Review Board of the Nepal Health Research Council (Reference number: 633/2021 P) on March 9, 2022. All Participants were informed and made aware about the research objectives, ensuring voluntary participation and privacy. All participants provided written informed consent. For face-to-face interviews, written consent was obtained on-site. For telephone interviews, we obtained both written consent via email and verbal consent during the calls. Initially, we collected information from the Ministry of Health and Population (MoHP) website to create a list of hospitals that have ICU facilities ^18^. Since the available information was incomplete, we included data from the website “CovidNepal” ^19^ to supplement the list. Additionally, we collaborated with the Nepal Health Research Council to ensure the accuracy and comprehensiveness of the list. We then excluded hospitals that had ICUs in the past but were not operational at the time of our survey. As a result, a total of 110 hospitals equipped with ICU facilities were identified in Bagmati Province. To proceed with the survey, we contacted the ICU representative of each hospital via email or telephone. Unfortunately, we were unable to establish contact with four hospitals, while 19 hospitals declined to participate. As a result, data from 87 eligible hospitals were included in the survey.

From the 87 hospitals, a total of 123 mixed medical/surgical ICUs were identified. 123 is the result from a hospital with 2 or more types of ICU. For the interviews, we selected one healthcare worker per ICU, such as the chief, in charge, or the most responsible person. These interviews involved various professionals, including nurses, physicians, allied health practitioners, administrators, and managers. Face-to-face interviews were done for 94 ICU facilities while telephone interviews were conducted for 29 sites, with each interview lasting around 20 to 30 minutes.

All interviews were audio-recorded. To avoid potential self-reporting biases associated with telephone interviews, we combined interview information with other sources, such as hospital records wherever possible. Most importantly, we encouraged participants to provide honest and anonymous feedback, assuring them that their responses will not impact their professional status or relationships. Regular data validation exercises were conducted to identify inconsistencies or outliers. The survey was conducted between 20th June and 26th November 2022.

The data collection tools used in this study ^20^ were adapted from the previously published survey conducted in Sri Lanka ^7^. The original survey in Sri Lanka focused on four main areas related to critical care units (CCUs): demography of CCUs, availability of facilities for monitoring and treatment, staffing composition, and patient characteristics and outcomes. The tools were modified and customized to suit the specific context and objectives of the study conducted in the Bagmati province of Nepal ^20^. A few additional changes were made after consulting with the local stakeholders. General service readiness of the ICUs was measured based on facility characteristics in five domains: 1) Access and organizational structure, 2) Infrastructure and essential equipment, 3) Human resources, team structure, and training opportunities, and 4) ICU service activities, protocols, and policies. The survey was designed using Research Electronic Data Capture ( REDCap), a secure web-based platform. Pilot testing of the survey questions was conducted in five hospitals, and feedback from the pilot and clinical sensibility testing was used to further refine the questions.

The study adheres to the reporting standards of the Strengthening the Reporting of Observational Studies in Epidemiology (STROBE) Statement and STROBE checklist for cross-sectional studies ^21^. Data collected during the survey ^20^ were entered into a Microsoft Excel version 16.0, and the data analysis was performed using descriptive statistics which involved means, medians, frequencies, and percentages.

## Results

### Domain 1: Access and organizational structure

**Figure 1.**
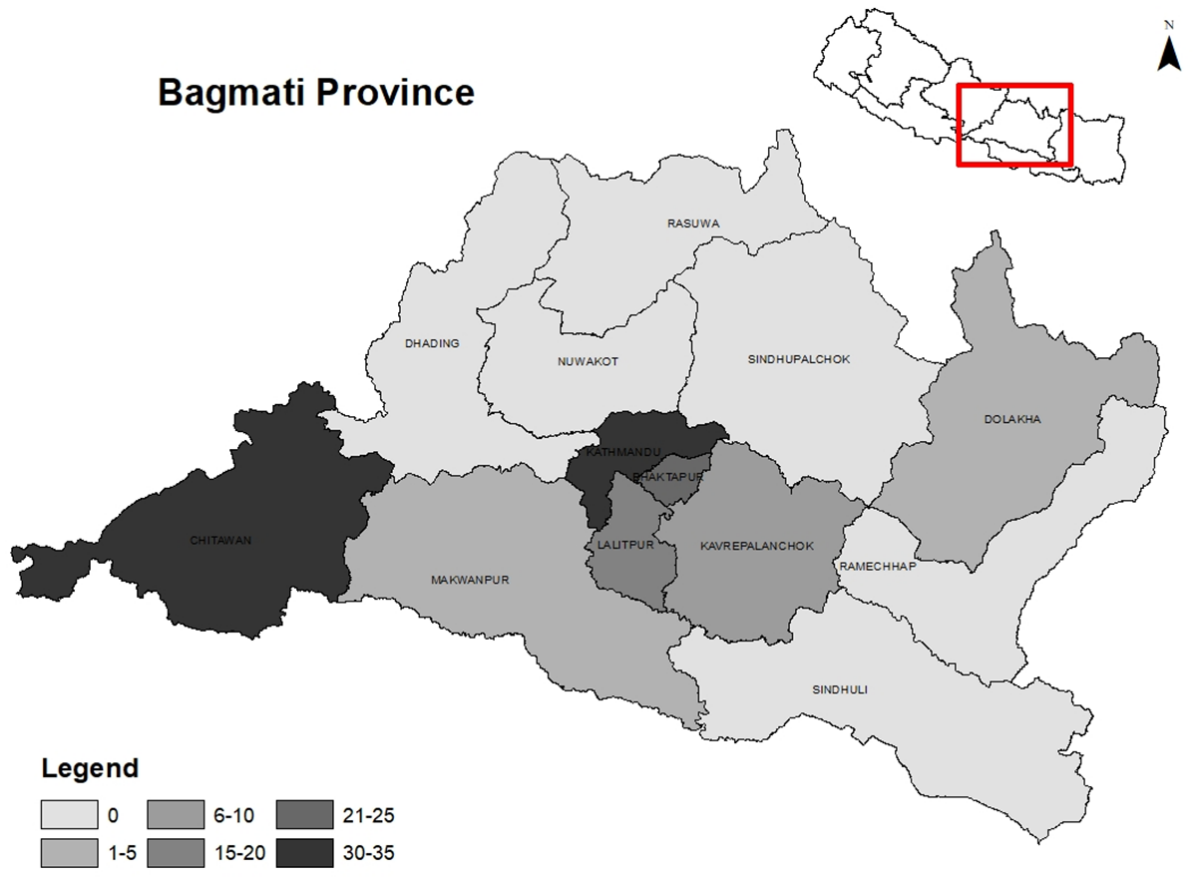
ICU distribution by administrative regions.

Access in our study referred to the geographic availability and distribution of ICU facilities, including ICU beds and ventilated beds across the 13 districts of Bagmati Province. A total of 123 ICU facilities providing 1167 ICU beds were identified in the province ( Table 1). Among the 13 districts, six districts did not have any ICUs. More than half of the ICUs (57.7%) were concentrated in Kathmandu, followed by Chitwan that accounted for one-fifth (21.1%) of the total ICU facilities. The distribution of ICU beds by administrative regions is displayed in Figure 1. The average number of ICU beds within hospitals per 100,000 population was 19, ranging from 3.6 in Makwanpur to 33.9 in Kathmandu. The median number of critical care beds per unit was 9 with an interquartile range of 6 to 10. Out of 1167 ICU beds, 692 were designated for ventilated patients, but the number of functioning ventilators was only 615. Regarding ownership ( Table 2), the majority of ICUs 86 (69.9%) were managed by private entities, while government institutions and non-profit entities managed 26.8% and 3.3%, respectively. In terms of the model of care, less than one-third 34 (27.6%) of the ICUs reported implementing a closed model of care. Furthermore, in terms of ICU distribution by type, visiting policy, and affiliation ( Table 3), the majority of ICUs (78.9%) were capable of caring for medical, surgical, and mixed/general cases, and only two units (1.6%) allowed unrestricted visiting time for families. Regarding affiliation, one-fourth of the hospitals (25.2%) were affiliated with a university, and only two hospitals were recognized by the professional body for intensive care medicine training.

**Table 1.** Distribution of beds and ventilators across districts of Bagmati province.

Districts in Bagmati Province	No of ICU (N=123)	No of ICU beds (N=1167)	Ventilated beds (N= 692)	No of ventilators (N= 615)	Population (2021 census survey)	ICU Beds per 100,000 population
Kathmandu	71 (57.7)	693 (59.4)	475 (68.6)	408 (66.3)	2,041,587	33.9
Bhaktapur	9 (7.3)	92 (7.9)	35 (5.1)	30 (4.9)	432,132	21.3
Lalitpur	11 (8.9)	103 (8.8)	67 (9.7)	57 (9.3)	551,667	18.6
Chitwan	26 (21.1)	230 (19.7)	96 (13.9)	103 (16.7)	719,859	31.9
Dolakha	1 (0.8)	7 (0.6)	5 (0.7)	0 (0)	172,767	4.0
Kavrepalanchok	3 (2.4)	25 (2.1)	11 (1.6)	11 (1.8)	364,039	6.8
Makwanpur	2 (1.6)	17 (1.5)	3 (0.4)	6 (1)	466,073	3.6

ICU=intensive care unit, Median ICU beds [IQR] =9 [6–10] ICU Beds per 100,000 population in Bagmati province= 19 (Total population of Bagmati province is 6,116,866), 6 districts (Dhading, Nuwakot, Ramechhap, Rasuwa, Sindhuli, Sindhupalchok) had no ICUs during our survey period. Hospitals that rejected to participate in the survey were from Kathmandu (14 hospitals), Lalitpur (4 hospitals) and Bhaktapur (1 hospital).

**Table 2.** No of ICU beds, ventilation and oxygenation capacity across different institution.

Institution category	No of beds (N=1167)	Beds capable of invasive ventilation (N=692)	Functioning ventilators (N=615)
Government (n=33)	346 (29.6)	246 (35.5)	203 (33)
Private (n=86)	792 (67.9)	429 (62)	396 (64.4)
Not for profit Hospital (n=4)	29 (2.5)	17 (2.5)	16 (2.6)
**Model of care**			
Open Unit (n=68 )	584 (50)	349 (50.4)	312 (50.7)
Closed Unit (n=34)	365 (31.3)	213 (30.8)	188 (30.6)
Semi closed Unit (n=21)	218 (18.7)	130 (18.8)	115 (18.7)

ICU=intensive care unit, 1:1 Ventilator-to-Bed Ratio = 692 / 1167 =0.59 Open units were defined as units where any attending physician can be the physician of record and can direct ICU care. Closed Units were defined as units having a dedicated intensivist responsible for overseeing the care of all patients in the ICU and having a defined care team 24 hours a day. Semi-closed units, also called hybrid/transitional ICU were defined as the one in which patients are co-managed by both the admitting physician and an intensivist-led team ^22^.

**Table 3.** ICU Distribution by type, visiting policy, and affiliation.

Characters	N (%) (N =123)
**Type of ICU**	
General/Mixed ICU	61 (49.6)
Medical ICU	24 (19.5)
Surgical ICU	12 (9.8)
Specialized *	26 (21.1)
**Visiting policy**	
Anytime	2 (1.6)
Hospital visiting time	17 (13.8)
Specified by ICU	104 (84.6)
**Hospital affiliation**	
Affiliated with a University	31 (25.2)
Affiliated with a Medical Education Commission (MEC)	12 (9.8)
Recognized by professional body for residency training	8 (6.5)
Recognized by professional body for intensive care medicine training	2 (1.6)
Recognized by professional body for critical care nursing training	1 (0.8)
None of the above	69 (56.1)

ICU=intensive care unit, N=Number of ICU, *Specialized ICUs included the ICUs capable of caring for neonatal, pediatric, neurological, nephrological, urological, post-operative and Covid-19 cases.

### Domain 2: Infrastructure and essential equipment

Table 4 presents information about the available infrastructure and facilities in the ICUs. A significant proportion of ICUs possessed essential resources such as piped oxygen (89.4%), wall suction units (81.3%), piped medical air (70.7%), and backup generators (86.2%). Essential equipment like syringe pumps (95.9%) and infusion pumps (96.7%) were widely accessible. However, some resources, including oxygen plants (37.4%) and extracorporeal membrane oxygenation (ECMO) machines (3.3%), were less commonly found. Hand washing facilities (95.1%) and access to communication tools like the internet (82.1%) and telephone (79.7%) were generally available. Notably, isolation rooms were present in less than half of the ICUs (46.3%). The table also indicates the distribution of arterial blood gas (ABG) machines, with 73.7% located within the ICU or in the main hospital laboratory (18.9%). Monitoring capabilities varied, with non-invasive monitoring being more prevalent (74%) compared to invasive arterial monitoring (45.5%). Additionally, capnography was present in 35.8% of ICUs, and cardiac output monitors were available in 22% of ICUs.

**Table 4.** ICU distribution by available infrastructure and facilities.

Infrastructure and equipment	N (%) (N =123)
**ICU equipment**	
Piped oxygen	110 (89.4)
Wall suction units	100 (81.3)
Piped medical air	87 (70.7)
Syringe pumps	118 (95.9)
Infusion pumps	119 (96.7)
Difficult Airway Trolley (DAT)	104 (84.6)
Oxygen plant	46 (37.4)
Backup generator availability	106 (86.2)
Hand washing facilities	117 (95.1)
Isolation room	57 (46.3)
Access to telephone	98 (79.7)
ECMO machine	4 (3.3)
Access to internet	101 (82.1)
Access to ABG machine	95 (77.2)
**Location of ABG machine**	
Main hospital laboratory	18 (18.9)
Another ICU	7 (7.4)
Within ICU	70 (73.7)
**Monitoring facilities availability**	
ABG machine measuring Hb	84 (68.3)
Invasive arterial monitoring	56 (45.5)
Noninvasive monitoring	91 (74)
Capnograph	44 (35.8)
Cardiac output monitors	27 (22)

*N=Number of ICU, ICU=intensive care unit, ECMO=extracorporeal membrane oxygenation machines, ABG=arterial blood gas.*

### Domain 3: Human resources, team structure, and training opportunities

Table 5 presents information about the distribution of human resources within the ICU. Anesthesiologists were the chief managers in just over a third (36.6%) of the ICUs, while internal medicine specialists were present in 21.1%. Notably, the majority of ICUs (85.3%) had registered nurses as primary in charge. In terms of patient care, a 1:1 nursing ratio was maintained for ventilated cases, both during the day (63.4%) and at night (62.6%). However, the nursing ratio for non-ventilated cases was considerably lower, with only 13 % of ICUs adhering to the 1:1 ratio. A significant number of ICUs had non-consultant doctors without other work commitments throughout the day (78%) and night (75.6%). The availability of other paramedical and support staff was also evident, with dedicated physiotherapists (52%), nutritionists (30.1%), clinical pharmacists (42.3%), and radiology technicians for portable X-rays (95.1%).

**Table 5.** ICU distribution by staffing and training.

Characters	N (%) N=123
**Speciality of ICU Head/Chief**	
Anesthesiologist	45 (36.6)
Internal medicine	26 (21.1)
Cardiologist	4 (3.3)
Pulmonologist	5 (4.1)
Emergency physician	5 (4.1)
Surgeon	5 (4.1)
Other	33 (26.8)
**In charge of ICU**	
Registered Nurse	105 (85.4)
Medical Officer	12 (9.8)
Paramedics *	6(4.9)
Availability of paramedical and support staff	
1:1 nursing of ventilated cases during day	78 (63.4)
1:1 nursing of ventilated cases during night	77 (62.6)
1:1 nursing of non-ventilated cases during day	16 (13)
1:1 nursing of non-ventilated cases during night	16 (13)
Non-consultant doctor with no other work commitment during day	96 (78)
Non-consultant doctor with no other work commitment during night	93 (75.6)
Supporting staffs per shift ^2^	60 (48.8)
Dedicated physiotherapist	64 (52)
Dedicated nutritionist	37 (30.1)
Dedicated clinical pharmacist	52 (42.3)
Radiology technicians for portable X-rays	117 (95.1)
**Training opportunities**	
Consultant In charge trained in critical care medicine (Yes) **	92 (74.8)
Nursing In charge trained in intensive care nursing (Yes)	48 (39)
Availability of ICU trained nurses (more than 5)	20 (16.3)
Doctors trained in Infection Prevention Control (more than 5)	7 (5.7)
Nurses trained in Infection Prevention Control (more than 5)	16 (13)

ICU=intensive care unit, N=Number of ICU, *Paramedics included Health Assistants and Technicians, **Consultants included critical care medicine specialists, general physician, surgeon, cardiothoracic surgeon, cardiologist, anesthesiologists, nephrologists, gynecologists, neurologists, psychiatrists and others, according to availability in descending order.

Furthermore, the table also provides insights into training opportunities within the ICU setting. A large proportion of ICU consultants 92 (74.8%) had received training in critical care medicine, resulting in one trained consultant per every 13 beds, or approximately 1.5 trained consultants per 100,000 population in the province. In contrast, the number of nurses in charge who have received training in intensive care nursing is comparatively lower 48 (39%), which makes one trained nurse in charge available for every 24 beds. Moreover, a small portion of both doctors (5.7%) and nurses (13%) had received training in infection prevention control.

### Domain 4: ICU service activities, protocols, and policies

Table 6 presents an overview of the activities, protocols, and policies implemented in the surveyed ICUs. A large majority of ICUs (79.7%) reported that they offer continuous medical education programs, and cardiopulmonary resuscitation (CPR) training (74%). Likewise, a substantial proportion of ICUs (69.1%) had quality improvement programs and conducted morbidity and mortality meetings (61.8%). However, only around one-third of ICUs mentioned carrying out clinical audits (30.9%), and engagement in research activities (28.5%). In terms of antibiotic usage, only 18.7% of the ICUs reported implementation of antibiotic stewardship programs. Regarding protocols, less than half (43.9%) of the ICUs had pain management protocols in place. Nevertheless, 66.7% of the ICU reported the implementation of infection control and prevention protocols.

**Table 6.** ICU distribution by activities, protocols and policies.

Activities	N (%)
Morbidity and mortality meeting	76 (61.8)
Quality Improvement programs	85 (69.1)
CPR training	91 (74)
Clinical audits	38 (30.9)
Continuous medical education	98 (79.7)
Research activities	35 (28.5)
Antibiotics stewardship programs	23 (18.7)
Other activities	6 (4.9)
**Protocol and policies**	
Pain management protocol	54 (43.9)
Nepalese Society of Critical Care Medicine ICU protocol	76 (61.8)
Infection control and prevention protocol	82 (66.7)

*N=Number of ICU*

## Discussion

This study was the first provincial-level survey in Nepal to assess the state of critical care services. While it should be noted that the study's results may not fully represent all ICUs in the Bagmati province due to the incomplete response rate of 87 out of listed 110 ICUs, they still provide valuable insights into the organizational structure, equipment availability, human resources, ICU activities, and policies, which hold significant implications for healthcare management and planning.

In terms of ICU bed capacity, the average number of beds per 100,000 population was found to be 19. Comparing this figure to the global average of 8.73 beds per 100,000 population in 87 countries ^23^, and to the national average of 2.8 ICU beds per 100,000 population in Nepal ^24^, this study highlights the higher availability of beds in the province. However, it is also important to consider that a country's hospital bed capacity is influenced by various factors ^25^ and this figure reflects the situation in Bagmati province where major urban areas, including the capital city, are located. Taking a closer examination of the districts in the Bagmati Province, there is a notable discrepancy in critical care capacity. Only four districts, namely Kathmandu, Bhaktapur, Lalitpur, and Chitwan, possess relatively higher critical care capacity (95%) compared to the remaining districts. Specifically, Kathmandu, being the capital city, stands out with nearly 60% of beds, indicating its ability to provide better critical care facilities for critically ill patients, especially those with respiratory distress. That being said, with a population of approximately 2.9 million residing in the remaining districts ^9^, it is a top priority at both the provincial and national levels to address this discrepancy in accessing critical care services ^26^.

Similarly, having an efficient referral system in place is also a critical aspect of ensuring timely access to intensive care services, especially when ICU beds are not available at the initial healthcare facility. In the context of Nepal, there is currently no formal referral mechanism within hospitals for transferring patients requiring ICU admission. When ICU beds are occupied, patients and their families are typically asked to find an available ICU on their own, which can be a significant challenge to accessing intensive care services, potentially leading to delays in treatment and adverse outcomes.

Another finding was that the majority of ICU beds, beds capable of invasive ventilation, and functioning ventilators were in private hospitals followed by government hospitals and not-for-profit hospitals. This dominance of private healthcare providers in critical care, which on average, are three to five times more expensive than government ICU ^15^, could lead to differences in how accessible and affordable critical care services are. One significant aspect to consider is the role of the government in establishing public ICUs and regulating them to ensure fair pricing and accessibility of critical care services. Similarly, in terms of the model of care, our study found that the highest number of beds, and mechanical ventilators are in the open units than the closed units, but in contrast, several studies have consistently shown that the closed model, which involves the active involvement of an intensivist, leads to reduced lengths of stay and mortality rates in critical care settings ^27– 32^. In addition to the bed capacity and model of care, the availability of essential equipment such as wall suctions, piped air, and piped oxygen was found to be relatively higher across Bagmati province. However, some gaps were identified in the availability of oxygen plants, isolation rooms and devices like cardiac output monitors. It is evident that the demand for these resources were heightened during Covid-19 pandemic ^33^, which highlights how crucial it is to have these resources readily available for future health crises. It is also equally important to consider the need for specialist knowledge and trained staff to ensure proper handling and utilization of these resources ^1, 7^. Therefore, it is imperative to prioritize strategies aimed at enhancing resource availability, improving infrastructure, and strengthening regulations.

Regarding the composition of human resources, our study noted just over a third of ICUs (36.6%) were managed by anesthesiologists. Unlike high-income countries, Nepal does not have a distinct specialty of intensive care medicine known as ‘intensivists’ or if available, they are scarce in number ^34^. However, our study revealed that trained consultants in critical care medicine (74.8%) were present with a ratio of one consultant per every 13 ICU beds, which falls within the recommended guideline range of a 1:8 -1:15 ratio ^35, 36^. Similarly, the nurse-patient ratio of 1:1 in more than 60% of the surveyed ICUs, along with the presence of paramedical and support staff, dedicated physiotherapists, nutritionists, and clinical pharmacists, indicates that the ICU is well-equipped to deliver high-quality care. However, there are areas of concern, such as limited training opportunities for nursing staff and infection prevention control. Lastly, this study also revealed the efforts undertaken by the majority of ICU facilities to raise the standard of patient care by engaging in different activities and protocols. The most commonly reported activities and protocols included Continuous Medical Education (CME), CPR training, infection control and prevention, pain management protocol, and ICU protocol. Nonetheless, there is still potential for development when it comes to conducting clinical audits, antibiotic stewardship programs and research projects.

Despite the presence of disparities, numerous ICUs were effectively carrying out multiple critical care procedures, aligning with findings from a comparable study conducted in the landlocked nation of Malawi ^37^. The information gathered from this study can be utilized by policymakers and healthcare providers to identify areas of need and allocate resources, accordingly, ensuring that critical care needs are adequately met in the Bagmati province. Regarding limitations, the study only focused on Bagmati province of Nepal limiting its generalizability to the broader context of the critical care resources and practices in the country. Not having the total bed count also limited our ability to calculate and report the desired ratio of ICU beds per hospital. Additionally, the study did not assess the quality of ICU care or patient outcomes, which are important factors in determining the effectiveness of critical care services. The study also did not look into other crucial care competencies of staff like patient safety, effective communication, and end-of-life care. Furthermore, the study did not look into the cost or affordability of critical care services, which is an important consideration in low- and middle-income countries like Nepal where financial barriers may limit access to care. Lastly, an incomplete response rate raises the possibility that the study's results may not fully reflect the state of all ICUs in the Bagmati province.

## Conclusion

This study sheds light on Bagmati Province’s critical care landscape highlighting both strengths and areas for improvement. The availability of critical care services in four districts was evident; however, there is a pressing need for a more balanced and decentralized approach to ensure equitable access to critical care services across the province. Additionally, the dominance of private healthcare entities in critical care highlights the importance of government intervention, public-private partnerships, and regulation to ensure fair access and affordability for all individuals. Policymakers and healthcare providers should prioritize identifying areas of need and allocating resources accordingly to ensure that critical care needs are met equitably. The study also emphasizes the need for infrastructure development, training opportunities, and research in improving the region's critical care quality. The limited research conducted in this area and the persistently poor outcomes emphasize the need for further investigations to improve patient care. It is imperative to upgrade future surveys to a nationwide scale, enabling a comprehensive assessment of critical care resources and practices across the country which would facilitate the development of national protocols and minimum standards to ensure consistent and high-quality care delivery.

## Data Availability

Figshare: Critical Care Services in Bagmati Province of Nepal: A Cross Sectional Survey. https://doi.org/10.6084/m9.figshare.24166557 ^20^. The project contains the following underlying data: critical_care_assessment_survey_data_2022.csv Figshare: Critical Care Services in Bagmati Province of Nepal: A Cross Sectional Survey. https://doi.org/10.6084/m9.figshare.24166557 ^20^. The project contains the following extended data: critical_care_assessment_survey_questionnaire.pdf Data are available under the terms of the Creative Commons Attribution 4.0 International license (CC-BY 4.0).
