# Supplementary material for: Critical care services in Bagmati province of Nepal: A cross sectional survey
Source: Wellcome Open Res. 2025 Jan 13;8:575. Originally published 2023 Dec 14. [Version 4] doi: 10.12688/wellcomeopenres.19932.4 (PMC11733805; doi:10.12688/wellcomeopenres.19932.4)
Supplement: Peer review history

# Reviewer report: Reviewer response for version 4

**DOI:** 10.21956/wellcomeopenres.26015.r117280

**Published:** 2025-01-18

**Authors:** Maria Srour

**Affiliations:** 1 Division of Pulmonary, Critical Care, Sleep, and Occupational Medicine, Indiana University School of Medicine, Indianapolis, Indiana, USA

Incredibly important information- well done! This provides much-needed information on the state of ICU availability and distribution in Nepal. Scientifically, I think this is a sound study and has been done well. I have a few citation issues to address and some comments on the presentation of results and discussion as below but overall think this is indexable.

**Citation issues:**

 The first two sources are specific to two countries, Pakistan and Uganda. The introduction may benefit from a more global discussion of critical care services in LMICs. They're older but one paper from Srinivas Murthy and another from Neill Adhikari give some nice estimates across LMICs. 

**Reference 25- **the paper currently reads "It is also important to consider that a country's hospital bed capacity is influenced by various factors ..." Would change to "It is also important to consider that a country's *needed* hospital and ICU bed capacity is influenced by various factors" based on the article being cited.

**Context:**

**Introduction:**

 Would also clarify what you mean by LMIC. The convention is to use LMIC for Low or Middle Income Country. Since Lower Middle is a specific economic distinction made by the World Bank, this use of LMIC might be confusing for readers.

**Methods:**

 no issues. survey looks comprehensive and was pilot tested

**Results:**

 How many hospitals in the province do not have an ICU? Are ICUs only available in tertiary or super specialty hospitals? Having this information might provide some further context about ICU availability and accessibility.

 The introduction mentions the 2014 mandate for 5% of total beds be ICU capable- may consider reporting in the results section or even in the second paragraph of the discussion how close the country is to achieving the 5% mandate.

 I am unsure of what the importance is of reporting what visitor hours are.

 One piece of data you have and don't report is the frequency with which the cardiac arrest team is called. Looks to be around 37% of the time. This may be interesting to report especially because you've mentioned CPR training as a metric. From a HIC, calling such a team or a 'code blue' is standard practice even in the ICU so this could be a very illustrative piece of information for readers in HICs to get an understanding of what ICU care in this context looks like.

**Discussion:**

 How many training programs (eg fellowships) are available for physicians and nurses hoping to do CCM in Nepal? If you have this information it might make the Human Resources part of your discussion more robust.

Is the work clearly and accurately presented and does it cite the current literature?

Partly

If applicable, is the statistical analysis and its interpretation appropriate?

Yes

Are all the source data underlying the results available to ensure full reproducibility?

Yes

Is the study design appropriate and is the work technically sound?

Yes

Are the conclusions drawn adequately supported by the results?

Yes

Are sufficient details of methods and analysis provided to allow replication by others?

Yes

Reviewer Expertise:

Critical care capacity in low resource settings

I confirm that I have read this submission and believe that I have an appropriate level of expertise to confirm that it is of an acceptable scientific standard, however I have significant reservations, as outlined above.

## References

[1] : Global health care of the critically ill in low-resource settings. *Ann Am Thorac Soc* .2013;10(5) : 10.1513/AnnalsATS.201307-246OT 509-13 10.1513/AnnalsATS.201307-246OT 24161054

[2] : Critical care and the global burden of critical illness in adults. *Lancet* .2010;376(9749) : 10.1016/S0140-6736(10)60446-1 1339-46 10.1016/S0140-6736(10)60446-1 20934212 PMC7136988

# Reviewer report: Reviewer response for version 4

**DOI:** 10.21956/wellcomeopenres.26015.r117145

**Published:** 2025-01-16

**Authors:** Jos Latour

**Affiliations:** 1 University of Plymouth Faculty of Health, Plymouth, England, UK

I suggest the manuscript to be accepted.

Is the work clearly and accurately presented and does it cite the current literature?

Yes

If applicable, is the statistical analysis and its interpretation appropriate?

Yes

Are all the source data underlying the results available to ensure full reproducibility?

Yes

Is the study design appropriate and is the work technically sound?

Yes

Are the conclusions drawn adequately supported by the results?

Yes

Are sufficient details of methods and analysis provided to allow replication by others?

Partly

Reviewer Expertise:

NA

I confirm that I have read this submission and believe that I have an appropriate level of expertise to confirm that it is of an acceptable scientific standard.

# Reviewer report: Reviewer response for version 4

**DOI:** 10.21956/wellcomeopenres.26015.r117144

**Published:** 2025-01-16

**Authors:** Simone Sandholz

**Affiliations:** 1 United Nations University, Bonn, Germany

I have no further comment on this paper. It has been revised accordingly

Is the work clearly and accurately presented and does it cite the current literature?

Partly

If applicable, is the statistical analysis and its interpretation appropriate?

Yes

Are all the source data underlying the results available to ensure full reproducibility?

Yes

Is the study design appropriate and is the work technically sound?

Yes

Are the conclusions drawn adequately supported by the results?

Partly

Are sufficient details of methods and analysis provided to allow replication by others?

Yes

Reviewer Expertise:

Critical infrastructures

I confirm that I have read this submission and believe that I have an appropriate level of expertise to confirm that it is of an acceptable scientific standard.

# Reviewer report: Reviewer response for version 4

**DOI:** 10.21956/wellcomeopenres.26015.r117140

**Published:** 2025-01-15

**Authors:** Mohan Gurjar

**Affiliations:** 1 Sanjay Gandhi Postgraduate Institute of Medical Sciences, Lucknow, India

No further comments.

Is the work clearly and accurately presented and does it cite the current literature?

Yes

If applicable, is the statistical analysis and its interpretation appropriate?

Yes

Are all the source data underlying the results available to ensure full reproducibility?

Yes

Is the study design appropriate and is the work technically sound?

Yes

Are the conclusions drawn adequately supported by the results?

Yes

Are sufficient details of methods and analysis provided to allow replication by others?

Yes

Reviewer Expertise:

Critical Care Medicine (Adult)

I confirm that I have read this submission and believe that I have an appropriate level of expertise to confirm that it is of an acceptable scientific standard.

# Reviewer report: Reviewer response for version 3

**DOI:** 10.21956/wellcomeopenres.25778.r116591

**Published:** 2025-01-10

**Authors:** Mohan Gurjar

**Affiliations:** 1 Sanjay Gandhi Postgraduate Institute of Medical Sciences, Lucknow, India

Authors presented a very useful aspect on the current status of critical care services in their region. They adequately presented the rationale for the current study.  The manuscript has appropriate background, methodology and result analysis, including tables and figures. The current version of the manuscript is acceptable and will add useful information in the literature,

Is the work clearly and accurately presented and does it cite the current literature?

Yes

If applicable, is the statistical analysis and its interpretation appropriate?

Yes

Are all the source data underlying the results available to ensure full reproducibility?

Yes

Is the study design appropriate and is the work technically sound?

Yes

Are the conclusions drawn adequately supported by the results?

Yes

Are sufficient details of methods and analysis provided to allow replication by others?

Yes

Reviewer Expertise:

Critical Care Medicine (Adult)

I confirm that I have read this submission and believe that I have an appropriate level of expertise to confirm that it is of an acceptable scientific standard.

# Reviewer report: Reviewer response for version 3

**DOI:** 10.21956/wellcomeopenres.25778.r116956

**Published:** 2025-01-06

**Authors:** Jos Latour

**Affiliations:** 1 University of Plymouth Faculty of Health, Plymouth, England, UK

Thank you for allowing me to review this well-written manuscript.

 I have one comments that needs clarification. In the methods section it is stated that interviews were conducted and recorded. The third paragraph in Method section starting with 'All interviews were recorded...'

 This reads as a qualitative study and so far my understanding is that this was a observational survey study. Can you please clarify what the interview guide was (was this the survey as described in the next paragraph?). I suggest to revise the paragraph and make it clear why interviews were performed, what interview guide was used (was it the survey) were the data collection tools closed or open-ended questions. Thus, is it that you used the survey you designed to collect data and with the survey you organised face to face meetings to go through all the items of the survey and complete all items (and you did the same with phone meetings?).

 For the rest I have no other comments.

 I commend the authors for the important work and the well-written manuscript.

Is the work clearly and accurately presented and does it cite the current literature?

Yes

If applicable, is the statistical analysis and its interpretation appropriate?

Yes

Are all the source data underlying the results available to ensure full reproducibility?

Yes

Is the study design appropriate and is the work technically sound?

Yes

Are the conclusions drawn adequately supported by the results?

Yes

Are sufficient details of methods and analysis provided to allow replication by others?

Partly

Reviewer Expertise:

Critical Care (ICU, PICU and NICU) and being a Trialist and  Methodologist with quantitative and qualitative research expertise

I confirm that I have read this submission and believe that I have an appropriate level of expertise to confirm that it is of an acceptable scientific standard, however I have significant reservations, as outlined above.

# Response

**Published:** 2025-01-06

**Authors:** Sushila Paudel

**Affiliations:** Nepal Intensive Care Research Foundation, Kathmandu, Central Development Region, Nepal

Dear Prof. Jos Latour,

 We appreciate your suggestion, which helped us improve the clarity and precision of the manuscript. We have taken them fully into account in revision. We are submitting the corrected manuscript with the suggestion to incorporate the manuscript in the track change mode. 

 To clarify, our study employed a structured survey questionnaire to collect data from ICU representatives. The term "interviews" was used to describe the administration of the survey via face-to-face and telephone methods. To address your concern, we have revised the methods section to make this distinction explicit. 

 The terms "face-to-face interviews" and "telephone interviews" have been clarified as structured sessions conducted to administer the survey. The term "interviews" was replaced with "survey" throughout the text to improve clarity. Additionally, the sentence "All interviews were audio recorded" has been revised to "All interviews conducted solely for administering the structured survey questionnaire were audio-recorded." The revised Methods section now addresses the  structured nature of the questionnaire, and the administration method, ensuring alignment with the study's cross-sectional survey design.

# Reviewer report: Reviewer response for version 2

**DOI:** 10.21956/wellcomeopenres.24778.r110474

**Published:** 2024-12-24

**Authors:** Emnet Tesfaye Shimber

**Affiliations:** 1 Hawassa University, Awasa, Ethiopia

It would have been good to have a general comparison of the countries critical care services with that of high income and low income countries. This allow us to see the major difference. It is better to mention the limitation in a separate title and shorten the conclusion. Also, better to include more recent references. Otherwise the paper is great.

Is the work clearly and accurately presented and does it cite the current literature?

Yes

If applicable, is the statistical analysis and its interpretation appropriate?

Partly

Are all the source data underlying the results available to ensure full reproducibility?

Yes

Is the study design appropriate and is the work technically sound?

Yes

Are the conclusions drawn adequately supported by the results?

Yes

Are sufficient details of methods and analysis provided to allow replication by others?

Partly

Reviewer Expertise:

Critical Care Medicine and Emergency Medicine

I confirm that I have read this submission and believe that I have an appropriate level of expertise to confirm that it is of an acceptable scientific standard.

# Reviewer report: Reviewer response for version 2

**DOI:** 10.21956/wellcomeopenres.24778.r104991

**Published:** 2024-11-19

**Authors:** Simone Sandholz

**Affiliations:** 1 United Nations University, Bonn, Germany

The study subject is a timely and relevant one. The paper is easy to follow and well-structured. I would have a few minor points that should be improved, that are fleshed out below. My only general reflection that I would recommend authors to revise is that the conclusions partly makes – very relevant – points that base on data not presented before. Hence I would recommend re-reading the conclusion to see if some of the data should still be added to the results part, before referring back to it in the conclusion chapter. That includes data on (functioning) equipment in private vs public hospitals, or

**Introduction:**

- First sentence: I believe these services are essential for any kind of country, but capacities differ a lot as fleshed out in the paper.
- The keywords says “lower middle income country” while the paper uses “low middle-income”, check for consistency, also Nepal is referred to as low middle…., while international statistics rank it lower middle?
- I would recommend adding a very few sentences around accessibility of hospitals and Nepal’s geography/road network, as these are crucial factors in this context, that would help readers understand the urgency of the research.

Methods

- Was the data obtained during interviews analysed “only” in the quantitative way described?

Results

- Regarding training, is there any information obtained how long ago such training was done, or if they are recurrent? That would be important information to have.

Is the work clearly and accurately presented and does it cite the current literature?

Partly

If applicable, is the statistical analysis and its interpretation appropriate?

Yes

Are all the source data underlying the results available to ensure full reproducibility?

Yes

Is the study design appropriate and is the work technically sound?

Yes

Are the conclusions drawn adequately supported by the results?

Partly

Are sufficient details of methods and analysis provided to allow replication by others?

Yes

Reviewer Expertise:

Critical infrastructures, sustainable urban and regional development

I confirm that I have read this submission and believe that I have an appropriate level of expertise to confirm that it is of an acceptable scientific standard, however I have significant reservations, as outlined above.

# Response

**Published:** 2024-12-20

**Authors:** Sushila Paudel

**Affiliations:** Nepal Intensive Care Research Foundation, Kathmandu, Central Development Region, Nepal

Dear Dr. Simone Sandholz, We appreciated the encouraging, critical and constructive comments on this manuscript. The comments have been very thorough and useful in improving the manuscript. We have taken them fully into account in revision. We are submitting the corrected manuscript with the suggestion to incorporate the manuscript in the track change mode. Our responses to all the comments are as follows:

 The study subject is a timely and relevant one. The paper is easy to follow and well-structured. I would have a few minor points that should be improved, that are fleshed out below. My only general reflection that I would recommend authors to revise is that the conclusions partly make – very relevant – points that base on data not presented before. Hence I would recommend re-reading the conclusion to see if some of the data should still be added to the results part, before referring back to it in the conclusion chapter. That includes data on (functioning) equipment in private vs public hospitals. 

**Response: **Thank you for your insightful comment. We have carefully reviewed the results and conclusion sections in response to your feedback. The results already describe the uneven distribution of ICU resources, the dominance of private healthcare providers, and the state of infrastructure and training opportunities. The conclusion builds on these findings by discussing their implications, emphasizing policy interventions, equitable resource distribution, and the need for further research. Additionally, we have made revisions to the conclusion to incorporate details on disparities in advanced equipment availability, such as ECMO machines, oxygen plants, and isolation rooms.

**Introduction:**

 First sentence: I believe these services are essential for any kind of country, but capacities differ a lot as fleshed out in the paper.

**Response: **We agree with your observation that critical care services are essential for any country, but the capacity indeed varies significantly across nations. We have revised the first sentence of the introduction to more clearly emphasize that critical care services are fundamental worldwide, but disparities in capacity exist, particularly in low- and middle-income countries, as outlined in the study.

 The keywords says “lower middle income country” while the paper uses “low middle-income”, check for consistency, also Nepal is referred to as low middle…., while international statistics rank it lower middle? 

**Response: **We have made the necessary adjustments to ensure consistency throughout the manuscript. The phrase “low and middle-income” has been standardized to “lower-middle-income” to align with international classification, and we have revised that Nepal is ranked as a lower-middle-income country in the context of global statistics.

 I would recommend adding a very few sentences around accessibility of hospitals and Nepal’s geography/road network, as these are crucial factors in this context, that would help readers understand the urgency of the research.

**Response: **Thank you for the suggestion. We have addressed this by adding few lines on the discussion of Nepal's mountainous terrain and inadequate road infrastructure create significant barriers to healthcare access.

****

**Methods**

 Was the data obtained during interviews analyzed “only” in the quantitative way described? **Response: **Thank you for your insightful question. A structured questionnaire was administered, and phone interviews were carried out as part of the data collection process. The data obtained through these methods were analyzed in a quantitative manner, using descriptive statistics such as means, frequencies, and percentages, as mentioned in the manuscript. We have not included qualitative aspects in this study, which is a limitation that we have acknowledged.

**Results**

 Regarding training, is there any information obtained how long ago such training was done, or if they are recurrent? That would be important information to have.

**Response: **Thank you for highlighting this important aspect. Unfortunately, our study did not collect specific information on the timing or recurrence of training sessions for ICU staff. We acknowledge that such data would provide valuable insights into the preparedness and continuous professional development of ICU personnel. We will consider incorporating this aspect into future studies to better understand the training dynamics and ensure a more comprehensive assessment of ICU capacity.

# Reviewer report: Reviewer response for version 2

**DOI:** 10.21956/wellcomeopenres.24778.r85729

**Published:** 2024-06-08

**Authors:** Mochamat Helmi

**Affiliations:** 1 Universitas Tarumanagara, West Jakarta, Jakarta, Indonesia

I have no further comment on this paper. It has been revised accordingly

Is the work clearly and accurately presented and does it cite the current literature?

Partly

If applicable, is the statistical analysis and its interpretation appropriate?

Yes

Are all the source data underlying the results available to ensure full reproducibility?

Yes

Is the study design appropriate and is the work technically sound?

Partly

Are the conclusions drawn adequately supported by the results?

Partly

Are sufficient details of methods and analysis provided to allow replication by others?

Partly

Reviewer Expertise:

Anesthesiology, Emergency, Intensive Care, Health care policy-management-quality, end of life care.

I confirm that I have read this submission and believe that I have an appropriate level of expertise to confirm that it is of an acceptable scientific standard.

# Reviewer report: Reviewer response for version 1

**DOI:** 10.21956/wellcomeopenres.22072.r75076

**Published:** 2024-05-11

**Authors:** Mochamat Helmi

**Affiliations:** 1 Universitas Tarumanagara, West Jakarta, Jakarta, Indonesia

Dear Author

 Thank you for your interesting writing. 

 This paper is aimed (as in abstract background and introduction): to assess 4 components, which are Access, infrastructure, human resources, and services.

 1. The introduction hasn't informed the background to support the aims, especially the access to the ICU. It is also not clear what access is referred to in this research.

 2. On the abstract it has been mentioned 87 mixed ICU while on the results it is mentioned 123 ICUs, I would suggest only to mention 123 as all results has been mentioned this number. However, a note must be made that 123 may results from a hospital with 2 or more type of ICU.

 3. The result, however, seemed aimed to show the ratio of availability on ICU beds per 100.000 population. It is rather more important to show the ratio how many beds available per hospital. This to indicate when there is an emergency case in the hospital, how ready is it to accommodate (in-house ICU).

 4. The paper also hasn't clarified the type of hospital. Are they primary / secondary / tertiary hospital. This mapping is very beneficial to understand the mapping. 

 5. Table 1 on my understanding remains under the issue of access. But there is already information about infrastructure (mechanical ventilation). I doubt this information is relevant at this section.

 6. What is the reason on displaying ECMO information o table 2 instead of other infrastructure data?

 7. Does the government have any guideline on an ideal number of ICU per hospital and per area?

 8. The paper showed interesting figure to map the availability. What about the access referral within hospitals? as it is an important part of access.

 9. Table 5. It is advised that the heading of the columns of this table is not ICU Chief primary specialty as the content do not inform merely only this information.

 10. Table 6, is there any data of other more crucial competencies such as: ICU training (clinical / managerial), resuscitation, basic / advanced life support, patient safety, effective communication, end of life care?

Is the work clearly and accurately presented and does it cite the current literature?

Partly

If applicable, is the statistical analysis and its interpretation appropriate?

Yes

Are all the source data underlying the results available to ensure full reproducibility?

Yes

Is the study design appropriate and is the work technically sound?

Partly

Are the conclusions drawn adequately supported by the results?

Partly

Are sufficient details of methods and analysis provided to allow replication by others?

Partly

Reviewer Expertise:

Anesthesiology, Emergency, Intensive Care, Health care policy-management-quality, end of life care.

I confirm that I have read this submission and believe that I have an appropriate level of expertise to confirm that it is of an acceptable scientific standard, however I have significant reservations, as outlined above.

# Reviewer report: Reviewer response for version 1

**DOI:** 10.21956/wellcomeopenres.22072.r72603

**Published:** 2024-01-25

**Authors:** Ana Maria Crawford

**Affiliations:** 1 Stanford University, Stanford, USA

This article sheds light on important issues in one province in Nepal and works through themes common to many resource-constrained settings.  It is methodologically sound and informative about the narrow objective it sets - ICU resources available in 1 province in Nepal. It makes a similar contribution to the increasing literature outlining the baseline needs for the implementation of critical care across healthcare systems.  It is commendable to continue to outline the problem of critical care for patients across the globe and particularly in settings suffering with resource-constraints, but the field must move forward in this endeavor mindful of the narratives and assumptions created along the way.  Continued focus on the patients and their needs is of utmost concern. 

 However, this article and many like it confound Intensive Care Units with Critical Care.  By focusing on ICU-level care and resources, we create a siloed perspective, doing a disservice to patients. In all settings, but particularly in resource-constrained settings, patients referred to ICU are often in extremis due to tardy recognition of their critical illness.  Many patients are never referred and expire elsewhere. Mortality rates in ICUs are therefore higher also lending higher and less efficient costs. ^1^ demonstrated that 90% of all critically ill patients are actually found outside of intensive care units. Critical illness occurs along a continuum and critical care must follow the patients, not the ICUs.

 Healthcare is about saving and improving the lives of patients. Without tracking clinical outcomes, infrastructure and interventions are moot. Quality of care is arguably more important than access to care in most settings. Most would rather a specialist trained in critical care manage their mechanical ventilator over an untrained nurse or even an untrained physician. Many may opt to not be intubated in the first place. Without skilled operators, equipment is ineffective and often harmful.

 Further, training must be better defined as assumptions are too often made about the workforce. Education and training vary widely across countries, specialties, and disciplines.  To have 'received critical care training; is vague. Without physician and nurse providers trained in the specialty of critical care medicine, patients will continue to enter ICUs with too few leaving alive, regardless of the presence of a mechanical ventilator and perhaps because of it.  Without the ability to recognize, respond, and rescue a patient from life-threatening reversible disease, the physical location of an ICU and all its equipment become a facade.

Is the work clearly and accurately presented and does it cite the current literature?

Yes

If applicable, is the statistical analysis and its interpretation appropriate?

Partly

Are all the source data underlying the results available to ensure full reproducibility?

Partly

Is the study design appropriate and is the work technically sound?

Partly

Are the conclusions drawn adequately supported by the results?

Partly

Are sufficient details of methods and analysis provided to allow replication by others?

Yes

Reviewer Expertise:

Anesthesiology, Critical Care Medicine, Global Health, Medical Education

I confirm that I have read this submission and believe that I have an appropriate level of expertise to confirm that it is of an acceptable scientific standard.

## References

[1] : The burden of critical illness among adults in a Swedish region-a population-based point-prevalence study. *Eur J Med Res* .2023;28(1) : 10.1186/s40001-023-01279-0 322 10.1186/s40001-023-01279-0 37679836 PMC10483802
